# Expression signatures of exosomal long non-coding RNAs in urine serve as novel non-invasive biomarkers for diagnosis and recurrence prediction of bladder cancer

**DOI:** 10.1186/s12943-018-0893-y

**Published:** 2018-09-29

**Authors:** Yao Zhan, Lutao Du, Lishui Wang, Xiumei Jiang, Shujun Zhang, Juan Li, Keqiang Yan, Weili Duan, Yinghui Zhao, Lili Wang, Yunshan Wang, Chuanxin Wang

**Affiliations:** 1 Department of Clinical Laboratory, The Second Hospital of Shandong University, Jinan, 250033 Shandong China; 2 Tumor Marker Detection Engineering Laboratory of Shandong Province, Jinan, Shandong China; 3 Department of Clinical Laboratory, Qilu Hospital of Shandong University, Jinan, 250012 Shandong China; 4 Department of Urology, Qilu Hospital of Shandong University, Jinan, 250012 Shandong China

**Keywords:** Bladder cancer, Urine exosomes, LncRNA, Non-invasive biomarkers, Diagnosis, Recurrence prediction

## Abstract

Recently, expression signatures of exosomal long non-coding RNAs (lncRNAs) have been proposed as potential non-invasive biomarkers for cancer detection. In this study, we aimed to develop a urinary exosome (UE)-derived lncRNA panel for diagnosis and recurrence prediction of bladder cancer (BC). Quantitative real-time polymerase chain reaction (qRT-PCR) was performed to screen and evaluate the expressions of eight candidate lncRNAs in a training set (208 urine samples) and a validation set (160 urine samples). A panel consisting of three differently expressed lncRNAs (MALAT1, PCAT-1 and SPRY4-IT1) was established for BC diagnosis in the training set, showing an area under the receiver-operating characteristic (ROC) curve (AUC) of 0.854. Subsequently, the performance of the panel was further verified with an AUC of 0.813 in the validation set, which was significantly higher than that of urine cytology (0.619). In addition, Kaplan-Meier analysis suggested that the up-regulation of PCAT-1 and MALAT1 was associated with poor recurrence-free survival (RFS) of non-muscle-invasive BC (NMIBC) (*p* < 0.001 and *p* = 0.002, respectively), and multivariate Cox proportional hazards regression analysis revealed that exosomal PCAT-1 overexpression was an independent prognostic factor for the RFS of NMIBC (*p* = 0.018). Collectively, our findings indicated that UE-derived lncRNAs possessed considerable clinical value in the diagnosis and prognosis of BC.

**Electronic supplementary material:**

The online version of this article (10.1186/s12943-018-0893-y) contains supplementary material, which is available to authorized users.

Bladder cancer (BC) is the most common malignancy of the urinary tract worldwide [1]. About 75% of patients are classified as non-muscle-invasive BC (NMIBC), which features a high recurrence rate. Moreover, roughly half of the muscle invasive BC (MIBC) patients will progress to metastasis and die within 3 years [2]. The poor prognosis of BC is partially due to lack of an effective means for early diagnosis. At present, the diagnosis of BC mainly relies on cystoscopy. However, the invasiveness of such procedure limit its use in mass cancer screening. Urine cytology has been proved to be ineffective as a tool to detect low-grade malignancy. Therefore, discovery of effective biomarkers for detection and recurrence prediction of BC can play pivotal roles in improving the prognosis of BC patients.

Exosomes are small vesicles with a diameter of approximately 30–150 nm. They are distributed in nearly all body fluids, including blood and urine. Exosomes can act as vehicles in cell-to-cell communication by transferring oncogenic molecules and play significant roles in tumorigenesis, progression and metastasis [3]. These functional contents are not stochastically packed into exosomes, which may rely on signature motifs, leading to the variation of exosomal contents under different pathological conditions or in different original cell types [4]. Studies have shown that BC cells can secrete exosomes into the urine and lncRNAs have been found to be stably present in exosomes [5]. Therefore, analyzing the expression profiles of urinary exosome (UE)-derived lncRNAs would provide valuable clues for diagnosis of BC. In this study, we systematically analyzed the expression profiles of UE-derived lncRNAs in BC patients and established a three-lncRNA panel for BC detection. Finally, we the explored the prognostic value of the selected exosomal lncRNAs.

## Results and discussion

### Characterization of UEs

**Fig. 1:**
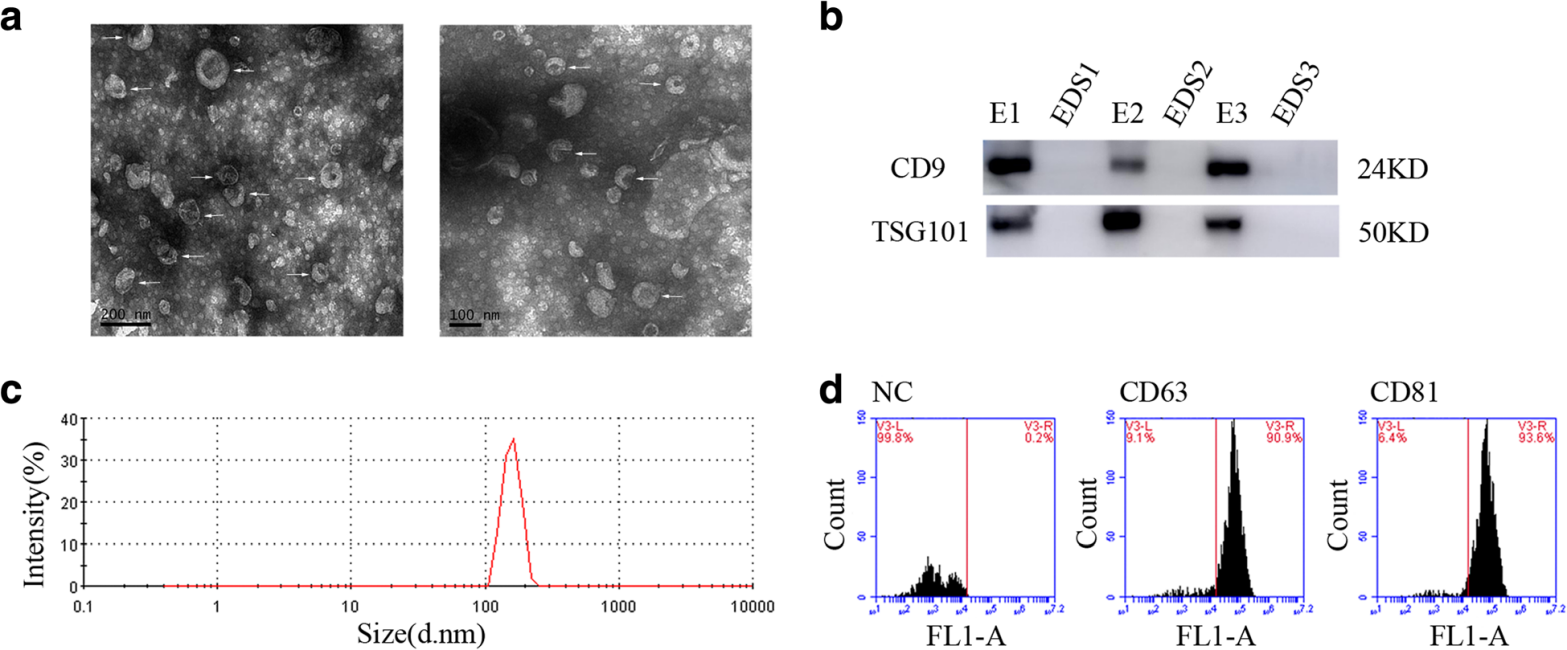
Characterization of UEs. **a** UEs were analyzed under TEM which exhibited a cup-shaped membrane morphology with a diameter of 60–150 nm. Typical exosomes were highlighted using white arrows. Left figure: scale bar = 200 nm; Right figure scale bar = 100 nm. **b** UEs-enriched protein markers including CD9 (24KD) and TSG101 (50KD) were analyzed by Western blotting in exosomes (E) and exosome-depleted supernatant (EDS). Three urine samples were used. **c** The sizes of urine exosomes were characterized via the NTA characterization system and the majority of vesicle particles were mainly between 60 and 150 nm in diameter. **d** Flow cytometry analysis was performed to detect the positive rate of CD63 and CD81 specific antibodies on the surface of exosomes

Transmission electron microscopy (TEM) showed UEs have a diameter of 60–150 nm with a cup-shaped membrane (Fig. 1a). Western blotting of UEs demonstrated the presence of CD9 and TSG101, which are exosome markers (Fig. 1b). Nanoparticle tracking analysis (NTA) found that particles ranging from 20 nm to 200 nm in diameter accounted for 98.1% (Fig. 1c). The flow cytometry showed that the positive rate of CD63 and CD81 specific antibodies on the exosome surface was 90.9% and 93.6%, respectively (Fig. 1d). Collectively, these data indicated that exosomes existed in urine, which laid a foundation for further study of exosomal biomarkers. The methods are explained in Additional file 1.

### Selection and evaluation of candidate UE-derived lncRNAs in BC patients

Eight lncRNAs (MALAT1, PCAT-1, SPRY4-IT1, UCA1, MEG3, H19, UBC1 and TUG1), which have been reported to play functional roles in tumorigenesis, were selected as candidate molecules [6–8]. Then, in the training set, the expressions of eight lncRNAs in 104 BC patients and 104 healthy controls were assessed by qRT-PCR. MALAT1, PCAT-1 and SPRY4-IT1 were significantly up-regulated in BC patients compared with the healthy controls (*p* < 0.001) (Additional file 3: Table S2) (Additional file 4: Figure S1 a-c).

To evaluate the performance of the identified lncRNAs for BC detection, we performed ROC curves in training set. The diagnostic accuracy of MALAT1, PCAT-1 and SPRY4-IT1, measured by AUC, was 0.844 (95% CI = 0.787 to 0.890, sensitivity = 72.1% and specificity = 84.6%), 0.832 (95% CI = 0.774 to 0.880, sensitivity = 72.1% and specificity = 81.7%) and 0.760 (95% CI = 0.696 to 0.817, sensitivity = 66.3% and specificity = 76.9%), respectively (Additional file 4: Figure S1 d-f). Subsequently, these lncRNAs were further verified in the validation set (80 BC patients and 80 healthy controls). The dysregulated expression trend was consistent between the two set (Additional file 3: Table S2). The corresponding AUCs of the three lncRNAs were 0.785 (95% CI =0.714 to 0.846, sensitivity =78.7% and specificity =67.5%), 0.810 (95% CI =0.741 to 0.868, sensitivity = 71.2% and specificity = 80.0%) and 0.799 (95% CI =0.728 to 0.858, sensitivity =87.5% and specificity =65%), respectively (Additional file 5: Figure S2).

### Analysis of the stability of identified lncRNAs in UEs

Next, two experiments were performed to verify the stability of UE-derived lncRNAs (MALAT1, PCAT-1 and SPRY4-IT1), considering that this is an essential prerequisite for biomarkers. Firstly, urine samples and exosome isolated nucleic acids were incubated with RNase A for 0, 30, 60 and 90 min. Strikingly, RNase A had no effect on the level of exosomal lncRNAs in the urine group (Additional file 6: Figure S3 a-c). However, the exosome isolated nucleic acids group was completely degraded by the treatment of RNase A within 30 min (Additional file 6: Figure S3 d-f). Secondly, urine samples were stored at − 80 °C for 1, 2 and 3 months. Results indicated that this treatment had no effect on the expression levels of MALAT1, PCAT-1 and SPRY4IT1 in UEs (Additional file 6: Figure S3 g-i). Collectively, our data indicated that the exosomal membrane can protect lncRNAs from being degraded, and their excellent stability makes exosomal lncRNAs ideal biomarkers for tumor diagnosis.

### Establishment of the UE-derived lncRNA panel for BC diagnosis

**Fig. 2:**
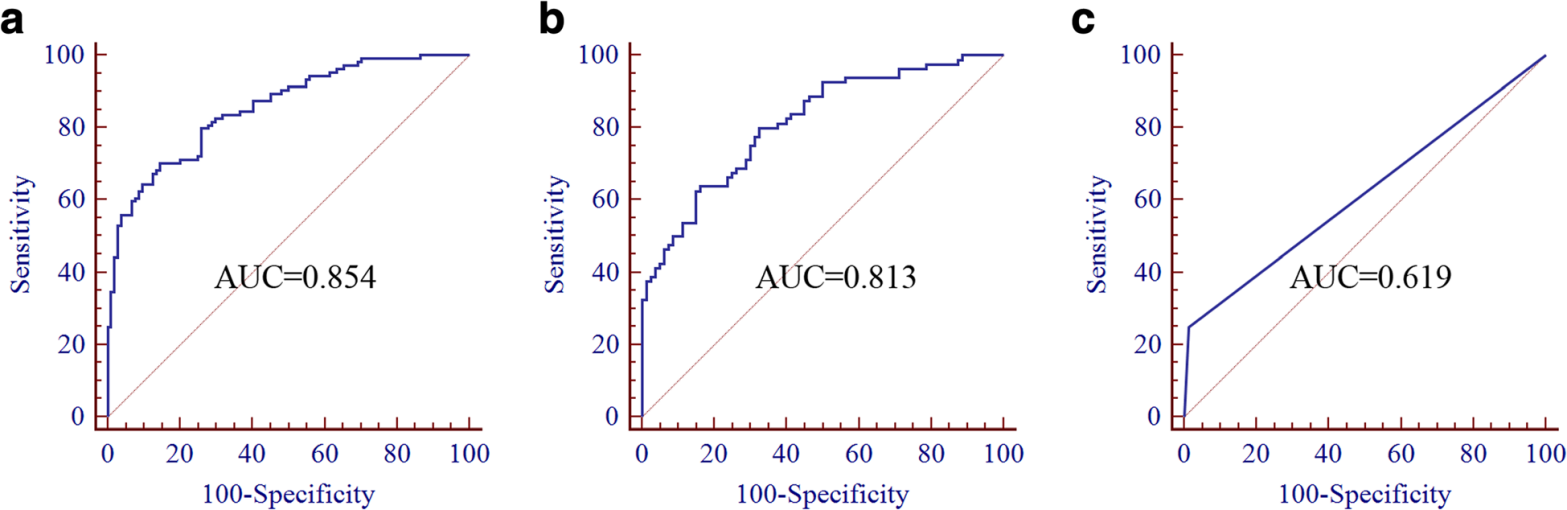
Evaluation the diagnostic performance of 3-lncRNA panel and urine cytology for BC diagnosis. ROC analysis was used to evaluate the performance of 3-lncRNA panel for the detection of BC in the training set (**a**) and in the validation set (**b**); ROC analysis revealed the diagnostic performance of urine cytology for BC diagnosis in the validation set (**c**)

Considering that combinations of tumor markers can improve the diagnostic accuracy, multivariate logistic regression model was performed in the training set to establish the selected exosomal lncRNA panel. The predictive probability of being diagnosed with BC was calculated using the equation as follows: Logit (*P*) = 0.6577–0.0695 × MALAT1–0.0686 × PCAT-1 – 0.0015 × SPRY4-IT1. The AUC of the panel was 0.854 (95% CI = 0.799–0.899, sensitivity = 70.2% and specificity =85.6%, Fig. 2a).

### Validation of the diagnostic performance of the lncRNA panel

To further verify the diagnostic performance of the panel, ROC analysis was performed in the validation set. Results showed that the AUC of the panel was 0.813 (95% CI = 0.744–0.870, sensitivity =62.5% and specificity = 85.0%) (Fig. 2b). Currently, urine cytology is widely used in clinical practice, but it has relatively poor sensitivity. Therefore, we compared the diagnostic performance between the panel and urine cytology. As expected, the AUC of urine cytology for BC detection was 0.619 (95% CI =0.539–0.694, sensitivity = 25% and specificity = 98.7%) (Fig. 2c), which was significantly lower than that of the panel.

### Correlation between the three UE-derived lncRNAs and clinicopathological characteristics

Next, we analyzed the correlation between the three UE-derived lncRNAs and clinicopathological characteristics of the BC patients. Results demonstrated overexpression of UE-derived PCAT-1 and SPRY4-IT1 were correlated with advanced TNM stage (all at *p* < 0.05). However, we did not find any significant association between the three lncRNAs and age, sex, tumor grade or positive lymph node metastasis (all at *p* > 0.05) (Additional file 7: Table S3).

### Correlation between the three UE-derived lncRNAs and recurrence-free survival (RFS)

**Fig. 3:**
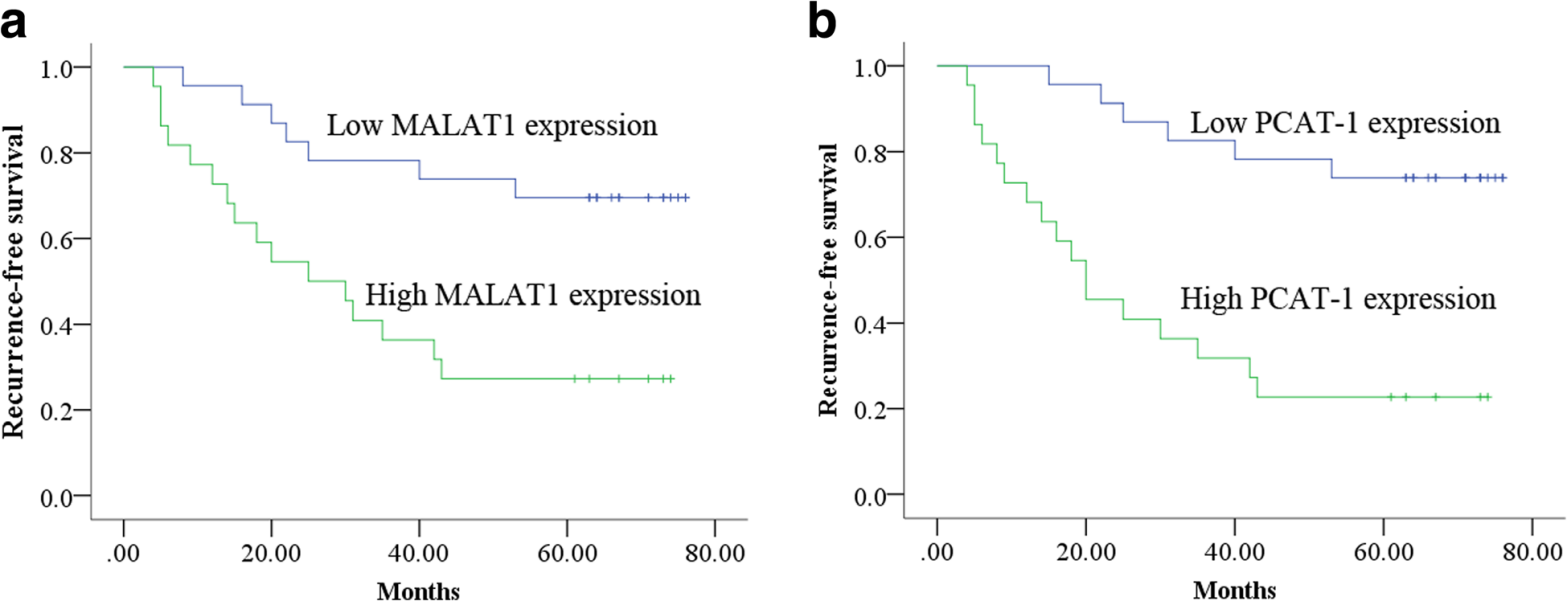
Recurrence prediction of UE-derived MALAT1 and PCAT-1 expression for NMIBC. Kaplan-Meier curve revealed that overexpression of UE-derived MALAT1 (**a**) and PCAT-1 (**b**) was relative to a poor recurrence-free survival in NMIBC patients from the validation set

To explore prognostic value of the three lncRNAs, BC patients were followed-up in the validation set. In the NMIBC group, results showed that patients with up-regulated MALAT1 and PCAT-1 had a significantly lower RFS (*p* = 0.002 and *p* < 0.001, respectively, Fig. 3a-b) compared with their corresponding counterparts. Afterwards, univariate Cox proportional hazards regression showed that there was a significant correlation between RFS of NMIBC and PCAT-1 (*p* = 0.001), MALAT1 (*p* = 0.005) or tumor stage (*p* = 0.001). Multivariate analysis revealed that PCAT-1 (*p* = 0.018) and tumor stage (*p* = 0.036) were independent prognostic factors for the RFS of NMIBC (Additional file 8: Table S4). However, none of the three lncRNAs were correlated with the recurrence of MIBC patients (all at *p* > 0.05, Additional file 9: Table S5).

Researchers have revealed UE-derived HOTAIR could serve as a biomarker for BC. However, their study are only conducted in a very small amount of urine samples, which are not verified in a larger population [9]. Moreover, considering that the progression of BC is a complex pathophysiological process, thus no single lncRNA can stand alone as a biomarker, but instead panel consisting of several lncRNAs will be necessary for BC diagnosis.

## Conclusions

We established a three-lncRNA panel for BC diagnosis through analyzing UE-derived lncRNAs, and identified that PCAT-1 could act as an independent risk factor for RFS of NMIBC. Further multi-center studies are necessary to verify the diagnostic efficiency of such panel before it could be adopted into clinical practice.

## Additional files

Additional file 1: Materials and Methods (Additional file 2: Table S1 and Additional file 10). (DOCX 17 kb)

Additional file 2: **Table S1.** Characteristics of the study population in the training set and the validation set. (DOCX 17 kb)

Additional file 3: **Table S2.** Expression of selected UE-derived lncRNAs in BC patients and healthy controls. (DOCX 14 kb)

Additional file 4: **Figure S1.** Concentrations of UE-derived MALAT1, PCAT-1 and SPRY4-IT1 and their diagnostic performance for detection of BC. Concentrations of UE-derived MALAT1 (a), PCAT-1 (b) and SPRY4-IT1 (c) in BCs (*n* = 104) vs. healthy controls (n = 104) using qRT-PCR assay in the training set (*P* < 0.001). ROC curve analysis showing the diagnostic performance for BC of UE-derived MALAT1 (d), PCAT-1 (e) and SPRY4-IT1 (f) in the training set. *** represents *P* < 0.001. (TIF 512 kb)

Additional file 5: **Figure S2.** Validation of UE-derived MALAT1, PCAT-1 and SPRY4-IT1 as biomarkers for BC diagnosis and their diagnostic performance. Concentrations of UE-derived MALAT1 (a), PCAT-1 (b) and SPRY4-IT1 (c) in BCs (*n* = 80) vs. healthy controls (n = 80) using the qRT-PCR assay in the validation set (*P* < 0.001). ROC curve analysis showing the diagnostic performance for BC of UE-derived MALAT1 (d), PCAT-1 (e) and SPRY4-IT1 (f) in the validation set. *** represents *P* < 0.001. (TIF 431 kb)

Additional file 6: **Figure S3.** Stability of UEs. Urine group and exosome isolated nucleic acids group were incubated with RNase A for 0, 30, 60, and 90 min, respectively. RNase A had no effect on the level of exosomal lncRNAs in urine group (a-c). However, exosome isolated nucleic acids group were completely degraded by the treatment of RNase A within 30 min (d-f). Urine samples were incubated at − 80 °C for 1, 2, and 3 months, and this treatments had no effect on the level of exosomal lncRNAs (g-i).* represents *P* < 0.05, ** represents *P* < 0.01, *** represents *P* < 0.001. (TIF 1232 kb)

Additional file 7: **Table S3.** Correlation between concentrations of UE-derived lncRNAs and clinicopathological characteristics of BC patients in the validation set. (DOCX 15 kb)

Additional file 8: **Table S4.** Univariate and multivariate Cox proportional hazards regression model analysis for prediction of RFS in NMIBC from the validation set. (DOCX 14 kb)

Additional file 9: **Table S5.** Univariate Cox proportional hazards regression model analysis for prediction of RFS in MIBC from the validation set. (DOCX 13 kb)

Additional file 10: Primer sequences. (DOCX 14 kb)

## Abbreviations

AUC: Area under the receiver-operating characteristic curve
BC: Bladder cancer
lncRNA: Long non-coding RNA
MIBC: Muscle invasive bladder cancer
NC: Negative controls
NMIBC: Non-muscle-invasive bladder cancer
NTA: Nanoparticle tracking analysis
qRT-PCR: Quantitative real time polymerase chain reaction
RFS: Recurrence-free survival
ROC: Receiver-operating characteristic
TEM: Transmission electron microscopy
TNM: Tumor-node metastasis
UE: Urinary exosome

## References

[1] Siegel RL, Miller KD, Jemal A (2018). Cancer statisticsx 2018. CA Cancer J Clin.

[2] Babjuk M, Bohle A, Burger M, Capoun O, Cohen D, Comperat EM (2017). EAU guidelines on non-muscle-invasive urothelial carcinoma of the bladder: update 2016. Eur Urol.

[3] Zhang X, Yuan X, Shi H, Wu L, Qian H, Xu W (2015). Exosomes in cancer: small particle, big player. J Hematol Oncol.

[4] Santangelo L, Giurato G, Cicchini C, Montaldo C, Mancone C, Tarallo R (2016). The RNA-binding protein SYNCRIP is a component of the hepatocyte exosomal machinery controlling microRNA sorting. Cell Rep.

[5] Beckham CJ, Olsen J, Yin PN, Wu CH, Ting HJ, Hagen FK (2014). Bladder cancer exosomes contain EDIL-3/Del1 and facilitate cancer progression. J Urol.

[6] Liu L, Liu Y, Zhuang C, Xu W, Fu X, Lv Z (2015). Inducing cell growth arrest and apoptosis by silencing long non-coding RNA PCAT-1 in human bladder cancer. Tumour Biol.

[7] Fan Y, Shen B, Tan M, Mu X, Qin Y, Zhang F (2014). TGF-β-induced upregulation of malat1 promotes bladder cancer metastasis by associating with suz12. Clin Cancer Res.

[8] Liu D, Li Y, Luo G, Xiao X, Tao D, Wu X (2017). LncRNA SPRY4-IT1 sponges miR-101-3p to promote proliferation and metastasis of bladder cancer cells through up-regulating EZH2. Cancer Lett.

[9] Berrondo C, Flax J, Kucherov V, Siebert A, Osinski T, Rosenberg A (2016). Expression of the long non-coding RNA HOTAIR correlates with disease progression in bladder cancer and is contained in bladder cancer patient urinary exosomes. PLoS One.

