# Supplementary material for: Expression signatures of exosomal long non-coding RNAs in urine serve as novel non-invasive biomarkers for diagnosis and recurrence prediction of bladder cancer
Source: Mol Cancer. 2018 Sep 29;17:142. doi: 10.1186/s12943-018-0893-y (PMC6162963; doi:10.1186/s12943-018-0893-y)
Supplement: Supplementary file 1 — Materials and Methods (Additional file 2: Table S1 and Additional file 10). (DOCX 17 kb) [file 12943_2018_893_MOESM1_ESM.docx]

**Methods**

**Study design**

In the present study, exosomes were firstly extracted from urine of BC patients and healthy controls and confirmed using transmission electron microscopy (TEM), Western blotting analysis, nanoparticle tracking analysis (NTA) and flow cytometry. Then, eight lncRNAs (MALAT1, PCAT-1, SPRY4-IT1, UCA1, MEG3, H19, UBC1 and TUG1) were selected as the candidate lncRNAs since their dysregulation in BC tissues and functional roles in tumorigenesis have been previously reported. Two sets of qRT-PCR were performed to analyze the expressions of candidate lncRNAs in UEs. All primers sequences involved in this study were showed in the additional files (Additional file 10). In the training set (including 104 BC patients and 104 healthy controls), three UE-derived lncRNAs were significantly up-regulated in BC patients compared with the healthy controls. Afterwards, the multivariate logistic regression model was used to construct a three-lncRNA diagnostic panel to differentiate BC patients from healthy people. Moreover, the stability of lncRNAs in UEs was investigated, considering that this is an essential prerequisite of a biomarker. In the validation set (including 80 BC patients and 80 healthy controls), the parameters of the logistic model were verified to validate the diagnostic performance of the established panel. In addition, the diagnostic performance between the three-lncRNA panel and urine cytology was also compared.

To explore whether these three lncRNAs had prognostic value for the recurrence of BC, BC patients in the validation set were followed up and assessed every 3 months during the first 2 years and thereafter every 6 months. The date of the latest record was February 28, 2018. The median follow-up time was 57 months (range of 4–76 months). A total of 45 NMIBC patients and 26 MIBC patients were applied to Kaplan-Meier survival analysis, while nine patients, including five with NMIBC and four with MIBC, were excluded because of incomplete information during follow-up.

**Patients and healthy controls**

Urine samples, which were chronologically allocated to the two sets, were collected from Qilu Hospital of Shandong University between 2012 and 2017. A total of 184 urine samples were obtained from BC patients, and 184 urine samples were obtained from healthy controls. The sample collection complied with the criteria as follows: urine samples from BC patients were collected before any antitumor therapies, such as surgery, chemotherapy or radiotherapy; urine samples of healthy controls were acquired from people who went through a medical check-up and showed no disease; and all these participants, with an age between 46 years and 88 years, showed no evidence of disease in other organs. Clinical features of BC patients and healthy controls were described in Additional file 2: Table S1.

The diagnosis of BC was performed based on histopathology or biopsy analysis. The tumor stage and grade complied with the tumor-node metastasis (TNM) staging system and the WHO 2004 grading scheme, respectively. The experimental protocol was approved by the Clinical Research Ethics Committee of Qilu Hospital, Shandong University, and informed consents were obtained from all participants prior to urine sample collection. All the procedures were performed in accordance with the ethical standards based on the Declaration of Helsinki (1964) and its later amendments.

**Exosome extraction and RNA extraction**

Midstream urine samples from BC patients and healthy controls were collected, centrifuged at 3,500 rpm for 5 min within 1 h, and further centrifuged at 10,000 rpm for 5 min to completely remove cell debris, and the supernatant fluids were then collected and stored at -80°C until exosome extraction.

Total exosomal RNA was extracted from the urine samples using the Urine Exosome RNA Isolation Kit (NORGEN, Product #47200) according to the manufacturer’s instructions and evaluated by a NanoDrop spectrophotometer (Thermo Fisher Scientific).

**qRT-PCR**

Purified RNA (1000 ng) was reversely transcribed into cDNA using the Prime Script™ RT Reagent Kit (Takara, Dalian, Liaoning, China) on a SimpliAmpTM Thermal Cycler (ABI, Singapore) according to manufacturer’s instructions. The reaction mixture was incubated at 37°C for 30 min, followed by 85°C for 5 s and 4°C for 60 min. qRT-PCR was performed on a CFX96 Real-Time PCR Detection System (Bio-Rad Laboratories, Hercules, CA, USA) using the SYBR Premix Ex Taq (Takara, Dalian, Liaoning, China). Briefly, after an initial denaturation step at 95°C for 30 s, the amplifications were carried out with 45 cycles at a melting temperature of 95°C for 5 s and an annealing temperature of 58°C for 34 s. The specificity of the PCR products was evaluated through melting curve analysis. Glyceraldehyde-3-phosphate dehydrogenase (GAPDH) was selected as the housekeeping gene, and the relative expressions of target genes were calculated by comparative cycle threshold (Ct) (2^−ΔΔCt^) method.

**Transmission electron microscopy (TEM)**

Exosomes extracted from urine samples were first resuspended in 200 µL PBS, and then a 20 µL aliquot was applied to a glow discharged 200-mesh Cu grid coated with carbon-Formvar film (ProSciTech, Kirwan, QLD, Australia) and allowed to absorb for 1 min. Finally, exosomes were stained with 20 µL 2% uranyl acetate at room temperature for 1 min and then dried using an infrared lamp for 10 min. Samples were imaged using a Tecnai G2 Spirit (FEI) TEM at 300 kV across 15,000 to 36,000 magnification.

**Western blotting analysis**

Total protein of UEs was extracted with 1x NuPAGE LDS Sample Buffer (Thermo Fisher Scientific) according to manufacturer’s instructions. Equal amounts of protein lysates were subjected to Western blotting analysis by using anti-CD9 antibody (rabbit IgG) (13174S, CST, USA), anti-TSG101 (mouse IgG) (Ab83, Abcam, UK), goat anti-rabbit HRP secondary antibody (ZB-2301, ZSGB-BIO, China) and goat anti-mouse HRP secondary antibody (ZB-2305, ZSGB-BIO, China) according to standard protocols as previously described. Immunoreactive bands were visualized using the Clarity Western ECL kit (Bio-Rad).

**Nanoparticle tracking analysis (NTA)**

The size distribution and concentration of exosomes were determined using NTA. Briefly, exosomes were diluted in 1 mL PBS and mixed well, and then the diluted exosomes were injected into the ZETASIZER Nano series-Nano-ZS instrument (Malvern, UK). Particles were automatically tracked and sized based on the Brownian motion and diffusion coefficient. Filtered PBS was used as a control.

**Flow cytometry**

Exosomal surface markers were determined using flow cytometry. Briefly, exosomes were resuspended in 100 µL PBS, and then the diluted exosomes were stained with FITC-conjugated CD63 antibody (BD, USA) and CD81 antibody (BD, USA). The unstained exosomes were used as negative controls (NC). Accuri C6 flow cytometer (BD, USA) was used to detect the exosomes according to the manufacturer’s instructions.

**Statistical analysis**

The differences in the expressions of UE-derived lncRNAs between BC patients and healthy controls were assessed by non-parametric Mann–Whitney U test. Scatter diagrams were made with GRAPHPAD PRISM 5 (San Diego, CA, USA). Logistic regression analysis was used through MATLAB software (MATLAB, R2014a, Natick, MA, USA) to establish the selected lncRNA panel. ROC curves and the area under the ROC curve (AUC) were employed to evaluate the diagnostic performance of the selected lncRNA panel in BC, and all of these analyses were performed on MEDCALC 15.2.2 (Med-Calc, Mariakerke, Belgium). Survival curves of NMIBC and MIBC patients were generated by Kaplan–Meier method, and the difference was compared by log-rank test. Cox regression analysis was used to identify independent prognostic factors for recurrence prediction. Statistical analysis was performed by SPSS STATISTICS 22.0 (IBM, Chicago, IL, USA). A *P* value < 0.05 was considered as statistically significant.
