# Supplementary material for: Expression signatures of exosomal long non-coding RNAs in urine serve as novel non-invasive biomarkers for diagnosis and recurrence prediction of bladder cancer
Source: Mol Cancer. 2018 Sep 29;17:142. doi: 10.1186/s12943-018-0893-y (PMC6162963; doi:10.1186/s12943-018-0893-y)
Supplement: Supplementary file 2 — Table S1. Characteristics of the study population in the training set and the validation set. (DOCX 17 kb) [file 12943_2018_893_MOESM2_ESM.docx]

**Table S1:** Characteristics of the study population in the training set and the validation set

| **Variable** | **Training set** | **Validation set** | ***P*-Value** |
| --- | --- | --- | --- |
|  | **n=208** | **n=160** |  |
| **Controls (number)** | 104 | 80 |  |
| **Age (years)** |  |  | 0.22 |
| ≤53 | 53 (50.96%) | 48 (60.00%) |  |
| >53 | 51 (49.04%) | 32 (40.00%) |  |
| **Sex** |  |  | 0.88 |
| Male | 79 (75.96%) | 60 (75.00%) |  |
| Female | 25 (24.04%) | 20 (25.00%) |  |
| **BC (number)** | 104 | 80 |  |
| **Age (years)** |  |  | 0.52 |
| ≤65 | 57 (54.81%) | 40 (50.00%) |  |
| >65 | 47 (45.19%) | 40 (50.00%) |  |
| **Sex** |  |  | 0.69 |
| Male | 82 (78.85%) | 65 (81.25%) |  |
| Female | 22 (21.15%) | 15 (18.75%) |  |
| **Tumor stage** |  |  | 0.60 |
| Ta-T1 | 61 (58.65%) | 50 (62.50%) |  |
| T2-T4 | 43 (41.35%) | 30 (37.50%) |  |
| **Tumor grade** |  |  | 0.54 |
| Low grade | 46 (44.23%) | 39 (48.75%) |  |
| High grade | 58 (55.77%) | 41 (51.25%) |  |
| **Lymph node metastasis** |  |  | 0.18 |
| Negative | 97 (93.27%) | 70 (87.50%) |  |
| Positive | 7 (6.73%) | 10 (12.50%) |  |

Abbreviations: BC, bladder cancer.
