# Supplementary material for: Expression signatures of exosomal long non-coding RNAs in urine serve as novel non-invasive biomarkers for diagnosis and recurrence prediction of bladder cancer
Source: Mol Cancer. 2018 Sep 29;17:142. doi: 10.1186/s12943-018-0893-y (PMC6162963; doi:10.1186/s12943-018-0893-y)
Supplement: Supplementary file 3 — Table S2. Expression of selected UE-derived lncRNAs in BC patients and healthy controls. (DOCX 14 kb) [file 12943_2018_893_MOESM3_ESM.docx]

**Table S2:** Expression of selected UE-derived lncRNAs in BC patients and healthy controls [median (interquartile range)]

| **LncRNA** | **Training set** | | |  | **Validation set** | | |
| --- | --- | --- | --- | --- | --- | --- | --- |
|  | **Controls (n=104)** | **BCs (n=104)** | ***p* Value** |  | **Controls (n=80)** | **BCs (n=80)** | ***p* Value** |
| MALAT1 | 0.99 (0.40-2.70) | 5.43(3.02-9.65) | < 0.0001 |  | 1.30(0.50-2.49) | 4.38(2.13-8.10) | < 0.0001 |
| PCAT-1 | 1.17 (0.38-3.27) | 6.39(2.81-12.18) | < 0.0001 |  | 1.16 (0.43-2.59) | 4.65(2.40-9.28) | < 0.0001 |
| SPRY4-IT1 | 1.21 (0.37-3.57) | 5.33(1.38-8.94) | < 0.0001 |  | 1.16 (0.42-3.76) | 5.48(2.65-10.40) | < 0.0001 |

Abbreviations: BC, bladder cancer; UE, Urinary exosome.
