# Supplementary figures and images for: Expression signatures of exosomal long non-coding RNAs in urine serve as novel non-invasive biomarkers for diagnosis and recurrence prediction of bladder cancer
Source: Mol Cancer. 2018 Sep 29;17:142. doi: 10.1186/s12943-018-0893-y (PMC6162963; doi:10.1186/s12943-018-0893-y)

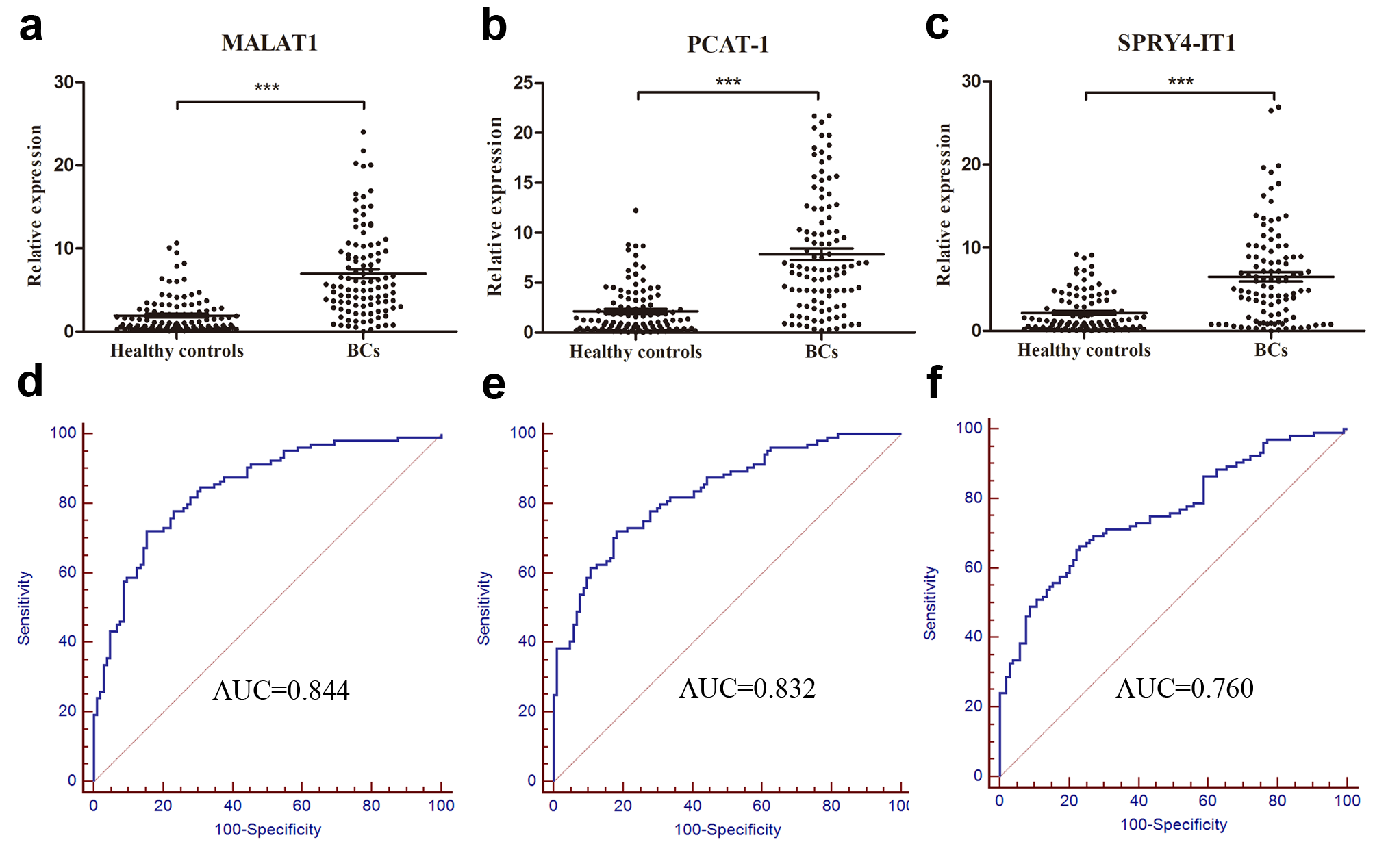

Supplement: Supplementary file 4 — Figure S1. Concentrations of UE-derived MALAT1, PCAT-1 and SPRY4-IT1 and their diagnostic performance for detection of BC. Concentrations of UE-derived MALAT1 (a), PCAT-1 (b) and SPRY4-IT1 (c) in BCs (n = 104) vs. healthy controls (n = 104) using qRT-PCR assay in the training set (P < 0.001). ROC curve analysis showing the diagnostic performance for BC of UE-derived MALAT1 (d), PCAT-1 (e) and SPRY4-IT1 (f) in the training set. *** represents P < 0.001. (TIF 512 kb) [file 12943_2018_893_MOESM4_ESM.tif]

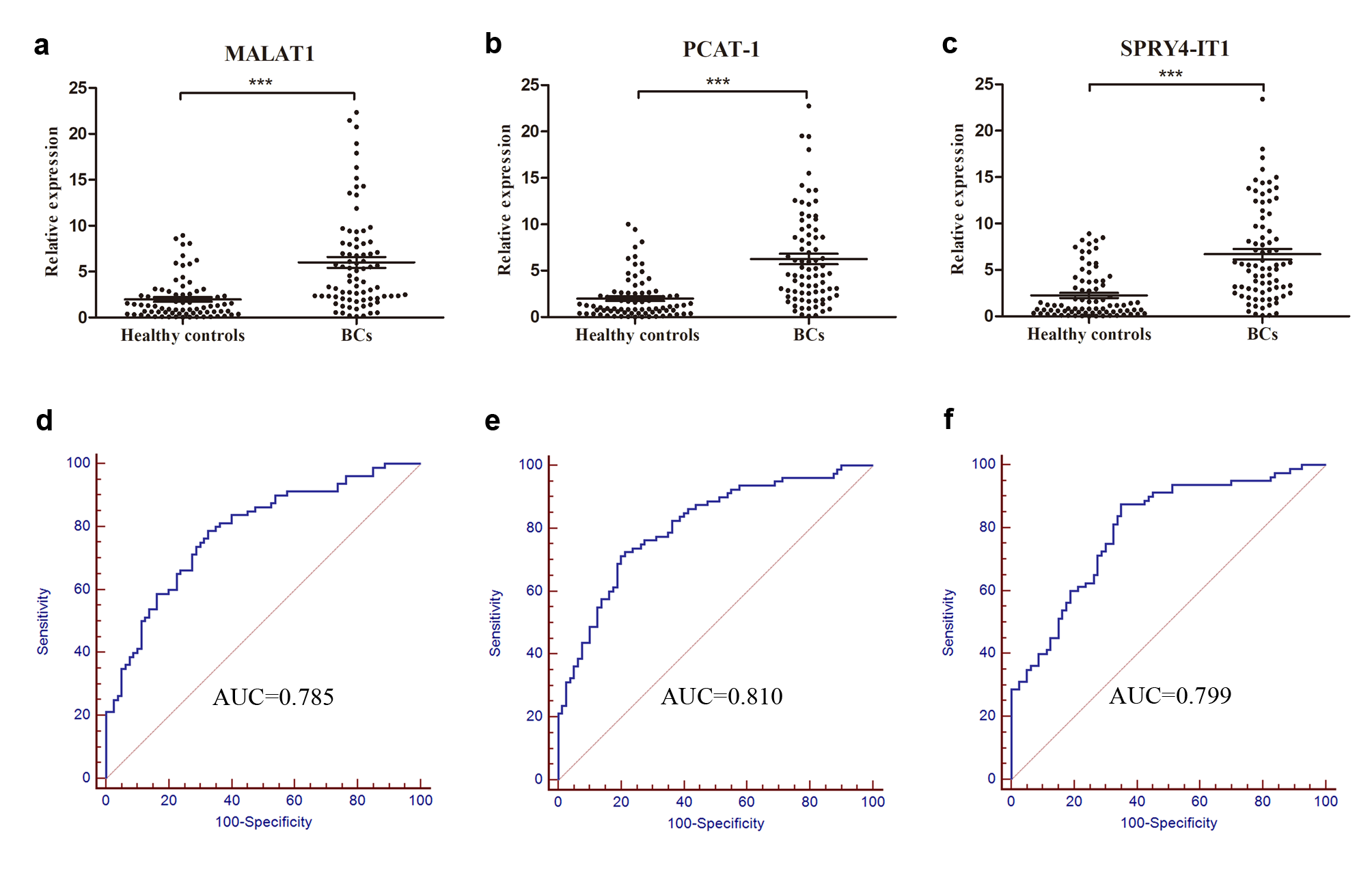

Supplement: Supplementary file 5 — Figure S2. Validation of UE-derived MALAT1, PCAT-1 and SPRY4-IT1 as biomarkers for BC diagnosis and their diagnostic performance. Concentrations of UE-derived MALAT1 (a), PCAT-1 (b) and SPRY4-IT1 (c) in BCs (n = 80) vs. healthy controls (n = 80) using the qRT-PCR assay in the validation set (P < 0.001). ROC curve analysis showing the diagnostic performance for BC of UE-derived MALAT1 (d), PCAT-1 (e) and SPRY4-IT1 (f) in the validation set. *** represents P < 0.001. (TIF 431 kb) [file 12943_2018_893_MOESM5_ESM.tif]

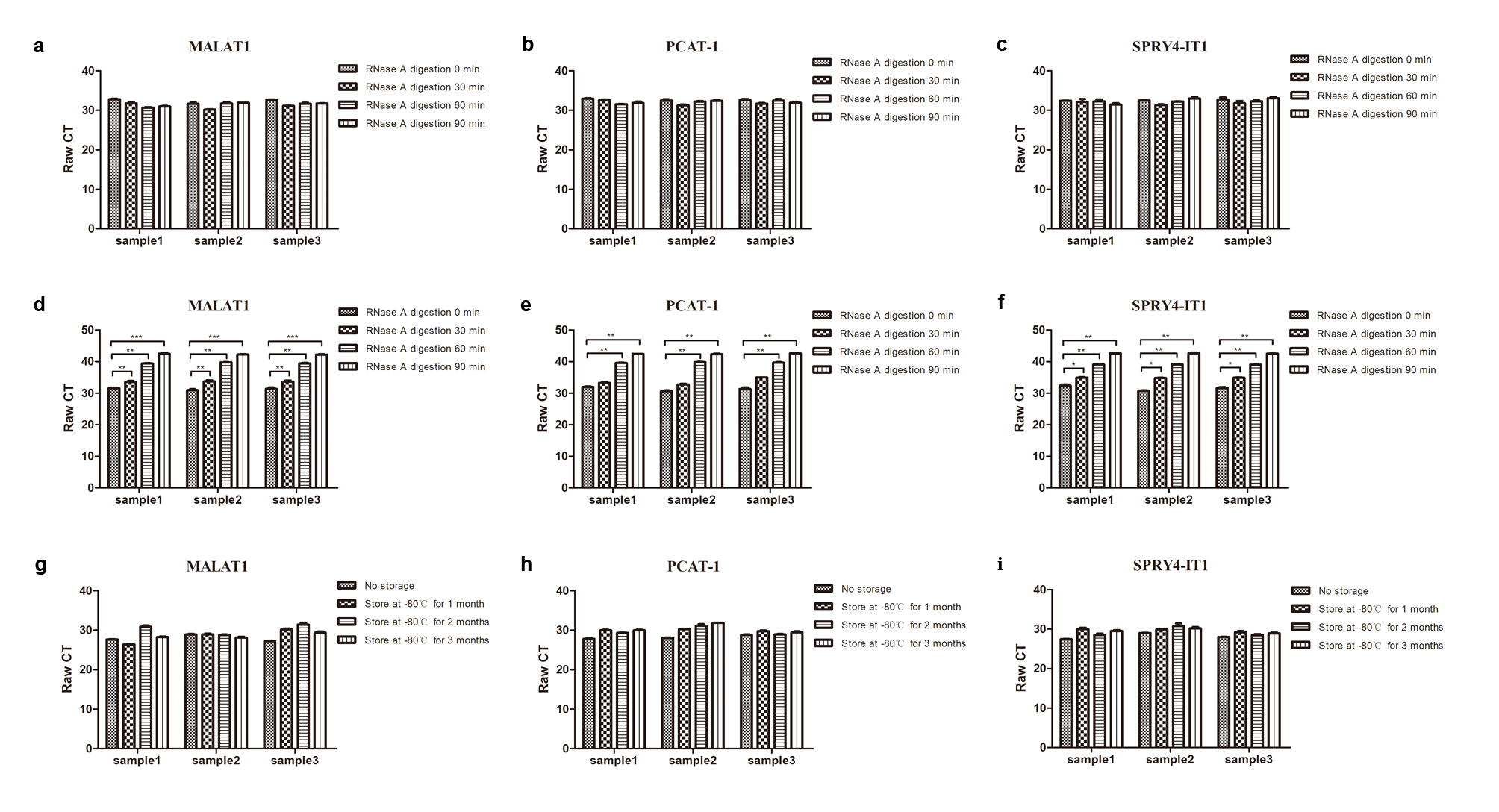

Supplement: Supplementary file 6 — Figure S3. Stability of UEs. Urine group and exosome isolated nucleic acids group were incubated with RNase A for 0, 30, 60, and 90 min, respectively. RNase A had no effect on the level of exosomal lncRNAs in urine group (a-c). However, exosome isolated nucleic acids group were completely degraded by the treatment of RNase A within 30 min (d-f). Urine samples were incubated at − 80 °C for 1, 2, and 3 months, and this treatments had no effect on the level of exosomal lncRNAs (g-i).* represents P < 0.05, ** represents P < 0.01, *** represents P < 0.001. (TIF 1232 kb) [file 12943_2018_893_MOESM6_ESM.tif]
