# Supplementary material for: Expression signatures of exosomal long non-coding RNAs in urine serve as novel non-invasive biomarkers for diagnosis and recurrence prediction of bladder cancer
Source: Mol Cancer. 2018 Sep 29;17:142. doi: 10.1186/s12943-018-0893-y (PMC6162963; doi:10.1186/s12943-018-0893-y)
Supplement: Supplementary file 7 — Table S3. Correlation between concentrations of UE-derived lncRNAs and clinicopathological characteristics of BC patients in the validation set. (DOCX 15 kb) [file 12943_2018_893_MOESM7_ESM.docx]

**Table S3:** Correlation between concentrations of UE-derived lncRNAs and clinicopathological characteristics of BC patients in the validation set [median (interquartile range)]

| **Parameters** | **Total cases** | **MALAT1** | ***P*** | **PCAT-1** | ***P*** | **SPRY4-IT1** | ***P*** |
| --- | --- | --- | --- | --- | --- | --- | --- |
| **Age** |  |  | 0.47 |  | 0.65 |  | 0.84 |
| ≤ 64 | 42 | 5.24 (2.17-10.60) |  | 4.59 (2.61-9.96) |  | 5.48 (2.43-10.36) |  |
| > 64 | 38 | 3.92 (2.08-7.25) |  | 4.92 (1.82-8.67) |  | 5.34 (2.79-10.73) |  |
| **Sex** |  |  | 0.21 |  | 0.28 |  | 0.39 |
| Male | 65 | 3.43 (1.99-8.08) |  | 4.49 (2.28-9.14) |  | 5.44 (2.34-9.67) |  |
| Female | 15 | 6.13 (2.76-8.26) |  | 5.53 (3.93-10.88) |  | 5.53 (3.18-14.46) |  |
| **Tumor stage** |  |  | 0.16 |  | 0.02 |  | 0.04 |
| Ta–T1 | 50 | 5.52 (2.36-8.31) |  | 5.99 (3.02-10.47) |  | 6.46 (2.96-12.33) |  |
| T2–T4 | 30 | 2.63 (1.78-7.34) |  | 3.07 (1.06-6.56) |  | 3.71 (1.81-7.04) |  |
| **Tumor grad**e |  |  | 0.84 |  | 0.46 |  | 0.37 |
| Low grade | 39 | 5.38 (2.26-8.05) |  | 5.38 (2.55-10.44) |  | 5.85 (2.88-11.05) |  |
| High grade | 41 | 3.90 (2.08-8.93) |  | 4.51 (2.03-8.61) |  | 4.46 (1.98-9.90) |  |
| **Lymph node metastasis** |  |  | 0.54 |  | 0.47 |  | 0.33 |
| Negative | 70 | 4.38 (2.05-7.75) |  | 4.65 (2.31-8.60) |  | 5.52 (2.87-11.36) |  |
| Positive | 10 | 5.53 (2.26-10.92) |  | 6.99 (2.46-12.21) |  | 4.13 (1.67-8.79) |  |

Abbreviations: BC, bladder cancer; UE, Urinary exosome.
