# Supplementary material for: Expression signatures of exosomal long non-coding RNAs in urine serve as novel non-invasive biomarkers for diagnosis and recurrence prediction of bladder cancer
Source: Mol Cancer. 2018 Sep 29;17:142. doi: 10.1186/s12943-018-0893-y (PMC6162963; doi:10.1186/s12943-018-0893-y)
Supplement: Supplementary file 8 — Table S4. Univariate and multivariate Cox proportional hazards regression model analysis for prediction of RFS in NMIBC from the validation set. (DOCX 14 kb) [file 12943_2018_893_MOESM8_ESM.docx]

**Table S4:** Univariate and multivariate Cox proportional hazards regression model analysis for prediction of RFS in NMIBC from the validation set

| **Parameters** | **Categories** | **Univariate analysis** | | **Multivariate analysis** | | |  |
| --- | --- | --- | --- | --- | --- | --- | --- |
|  |  | **HR(95%CI)** | ***P*-Value** | | **HR(95%CI)** | ***P*-Value** | |
| Age | ≤64 vs. >64 | 2.000(0.862-4.642) | 0.106 |  | |  |  |
| Sex | Male vs. Female | 1.447(0.569-3.680) | 0.438 |  | |  |  |
| Tumor stage | Ta vs. T1 | 4.641(1.814-11.874) | 0.001 | 3.547(1.087-11.570) | | 0.036 |  |
| Tumor grade | Low vs. High | 0.709(0.241-2.084) | 0.531 |  | |  |  |
| Lymph node metastasis | Negative vs. Positive | 1.230(0.288-5.256) | 0.780 |  | |  |  |
| MALAT1 expression | Low vs. High | 3.627(1.483-8.872) | 0.005 |  | |  |  |
| PCAT-1 expression | Low vs. High | 5.260(2.053-13.478) | 0.001 | 6.368(1.375-29.496) | | 0.018 |  |
| SPRY4-IT1 expression | Low vs. High | 1.990(0.859-4.605) | 0.108 |  | |  |  |

Abbreviations: RFS, Recurrence-free survival; NMIBC, Non-muscle-invasive BC; HR, Hazard ratio; CI, Confidence interval.
