# Supplementary material for: Expression signatures of exosomal long non-coding RNAs in urine serve as novel non-invasive biomarkers for diagnosis and recurrence prediction of bladder cancer
Source: Mol Cancer. 2018 Sep 29;17:142. doi: 10.1186/s12943-018-0893-y (PMC6162963; doi:10.1186/s12943-018-0893-y)
Supplement: Supplementary file 9 — Table S5. Univariate Cox proportional hazards regression model analysis for prediction of RFS in MIBC from the validation set. (DOCX 13 kb) [file 12943_2018_893_MOESM9_ESM.docx]

**Table S5:** Univariate Cox proportional hazards regression model analysis for prediction of RFS in MIBC from the validation set

| **Parameters** | **Categories** | **Univariate analysis** | |
| --- | --- | --- | --- |
|  |  | **HR(95%CI)** | ***P-*Value** |
| MALAT1 expression | Low vs. High | 1.943(0.630-5.990) | 0.247 |
| PCAT-1 expression | Low vs. High | 1.009(0.339-3.004) | 0.987 |
| SPRY4-IT1 expression | Low vs. High | 1.530(0.513-4.560) | 0.445 |

Abbreviations: RFS, Recurrence-free survival; MIBC, Muscle invasive BC; HR, hazard ratio; CI, confidence interval.
