# Supplementary material for: Expression signatures of exosomal long non-coding RNAs in urine serve as novel non-invasive biomarkers for diagnosis and recurrence prediction of bladder cancer
Source: Mol Cancer. 2018 Sep 29;17:142. doi: 10.1186/s12943-018-0893-y (PMC6162963; doi:10.1186/s12943-018-0893-y)
Supplement: Supplementary file 10 — Primer sequences. (DOCX 14 kb) [file 12943_2018_893_MOESM10_ESM.docx]

1. **MALAT1**

Forward primer 5’-AAAGCAAGGTCTCCCCACAAG-3’

Reverse primer 5’-GGTCTGTGCTAGATCAAAAGGCA-3’

1. **PCAT-1**

Forward primer 5’-GAGAGCTGACATAGGCACCC-3’

Reverse primer 5’-TCTCCACTGGTGTTCATGGC-3’

1. **SPRY4-IT1**

Forward primer 5’-CCCCAGAGAGCCAAGTCATC-3’

Reverse primer 5’-CACAGGCTCCCATAACCCTC-3’

1. **TUG1**

Forward primer 5’-CTGGACCTGGAACCCGAAAG-3’

Reverse primer 5’-GGTAGTGCTTGCTCAGTCGT-3’

1. **UBC1**

Forward primer 5’-ATCTCAAGGACTGGCGCTC-3’

Reverse primer 5’-AGACAGGACAAACTCCAGCC-3’

1. **H19**

Forward primer 5’-ATCTGGAGTCTGGCAGGAGTG-3’

Reverse primer 5’-AGCTGATGTCGCCCTGTCTG-3’

1. **MEG3**

Forward primer 5’-GGGCATTAAGCCCTGACCTT-3’

Reverse primer 5’-CCTTGGGGAGGGAAACACTC-3’

1. **UCA1**

Forward primer 5’-CTCTCCATTGGGTTCACCATTC-3’

Reverse primer 5’-GCGGCAGGTCTTAAGAGATGAG-3’

1. **GAPDH**

Forward primer 5’-ACCCACTCCTCCACCTTTGAC-3’

Reverse primer 5’-TGTTGCTGTAGCCAAATTCGTT-3’
